# Importance of appropriate genome information for the design of mating type primers in black and yellow morel populations

**DOI:** 10.1186/s43008-022-00101-6

**Published:** 2022-08-22

**Authors:** Melissa Cravero, Aaron J. Robinson, Patrick Hilpisch, Patrick S. Chain, Saskia Bindschedler, Pilar Junier

**Affiliations:** 1 Laboratory of Microbiology, Institute of Biology, University of Neuchâtel, 2000 Neuchâtel, Switzerland; 2 Bioscience Division, Los Alamos National Laboratory, Los Alamos, NM 87545 USA

**Keywords:** PCR, MAT idiomorphs, *Morchella*, Heterothallic, Homothallic, Sexual reproduction, Fruiting bodies, Morels

## Abstract

Morels are highly prized edible fungi where sexual reproduction is essential for fruiting-body production. As a result, a comprehensive understanding of their sexual reproduction is of great interest. Central to this is the identification of the reproductive strategies used by morels. Sexual reproduction in fungi is controlled by mating-type (*MAT*) genes and morels are thought to be mainly heterothallic with two idiomorphs, MAT1-1 and MAT1-2. Genomic sequencing of black (Elata clade) and yellow (Esculenta clade) morel species has led to the development of PCR primers designed to amplify genes from the two idiomorphs for rapid genotyping of isolates from these two clades. To evaluate the design and theoretical performance of these primers we performed a thorough bioinformatic investigation, including the detection of the MAT region in publicly available *Morchella* genomes and in-silico PCR analyses. All examined genomes, including those used for primer design, appeared to be heterothallic. This indicates an inherent fault in the original primer design which utilized a single *Morchella* genome, as the use of two genomes with complementary mating types would be required to design accurate primers for both idiomorphs. Furthermore, potential off-targets were identified for some of the previously published primer sets, but verification was challenging due to lack of adequate genomic information and detailed methodologies for primer design. Examinations of the black morel specific primer pairs (MAT11L/R and MAT22L/R) indicated the MAT22 primers would correctly target and amplify the MAT1-2 idiomorph, but the MAT11 primers appear to be capable of amplifying incorrect off-targets within the genome. The yellow morel primer pairs (EMAT1-1 L/R and EMAT1-2 L/R) appear to have reporting errors, as the published primer sequences are dissimilar with reported amplicon sequences and the EMAT1-2 primers appear to amplify the RNA polymerase II subunit (*RPB2*) gene. The lack of the reference genome used in primer design and descriptive methodology made it challenging to fully assess the apparent issues with the primers for this clade. In conclusion, additional work is still required for the generation of reliable primers to investigate mating types in morels and to assess their performance on different clades and across multiple geographical regions.

**Supplementary Information:**

The online version contains supplementary material available at 10.1186/s43008-022-00101-6.

## Background

True morels, belonging to the genus *Morchella*, are highly prized ascomycete fungi due to the exceptional organoleptic properties of their ascocarps. While morels can form ascocarps under appropriate environmental conditions in natural systems, production of these sexual structures through artificial cultivation remains a challenge (Liu et al. 2018a, b). This has motivated efforts to better understand the biological mechanisms that permit sexual reproduction in these fungi, with the aim of facilitating the cultivation of fruiting bodies. In heterothallic members of the Ascomycota, sexual reproduction is generally controlled by a bipolar mating system comprised of two mating idiomorphs most frequently identified as MAT1-1 and MAT1-2, although alternative identifiers are used in some cases (Robinson and Natvig 2019). Each idiomorph encodes a variable number of mating-type genes (Wilken et al. 2017) that produce unrelated proteins (Casselton 2002). Given that the genes at this locus have little homology, they are referred to as idiomorphs rather than alleles (Arie et al. 1997; Zheng et al. 2013; Chai et al. 2017). The genomes of monokaryotic individuals from heterothallic species possess only one of the two idiomorphs (either *MAT1-1* or *MAT1-2*) and they must find a partner with the opposite mating-type idiomorph to complete the sexual part of their life cycle and to produce ascospores (Coppin et al. 1997). In contrast, individuals from homothallic species are self-fertile as they either possess both mating-type idiomorphs within a single haploid genome (primary homothallism) or produce multinucleated spores with both mating idiomorphs present, but each in an individual haploid nucleus (secondary homothallism) (Wilson et al. 2015). An often-defining characteristic of the mating type idiomorphs are genes that contain high mobility group (HMG) domains. The *MAT1-1-1* gene encodes an α1 protein belonging to the MATα_HMG family, while *MAT1-2-1* encodes a protein belonging to the MATA_HMG family (Arie et al. 1997; Zheng et al. 2013; Zou et al. 2019; Robinson and Natvig 2019). In addition to their importance in determining the mode of reproduction of ascomycete fungi, mating-type genes can be a useful tool to improve species determination and phylogenetic analyses (Du et al. 2005). This is due to the observation that *MAT1-1-1* and *MAT1-2-1* have a high interspecific and low intraspecific variability in several examined fungal lineages (Coppin et al. 1997).

*Morchella* spp. are considered to primarily be heterothallic, but secondary homothallism (Du and Yang 2021) and primary homothallism (Chai et al. 2022) have also been observed. Furthermore, the mating strategies can be mixed within a single species. For instance, the black morel *Morchella importuna* can reproduce by three different mating systems: heterothallism, homothallism, and pseudohomothallism (Du and Yang 2021). However, no obvious morphological differences have been observed for fruiting-bodies and mycelia resulting from these various mating strategies. As a result, sequencing-based approaches and analyses are frequently utilized to characterize fungal mating strategies and genotypes. Once mating-type loci have been identified and sequenced for a particular fungal group or clade, it is quite common to design primers for the characterization of other closely related fungal isolates in a rapid and cost-efficient manner through the use of polymerase chain reaction (PCR) and gel electrophoresis (Du et al. 2017, 2020; Chai et al. 2017, 2019).

Primers designed to amplify partial regions of two *MAT* genes in *Morchella* spp. of the Elata (black) clade have been previously published (MAT11L/R and MAT22L/R; Du et al. 2017). Given that black and yellow morels differ in their ecology and morphology (Pilz et al. 2007) and are phylogenetically divergent (O’Donnell et al. 2011), the loci contributing to sexual mating could also be expected to differ. This was the motivation for the development of additional specific primer pairs to examine species of the Esculenta (yellow) clade as well (EMAT1-1 L/R and EMAT1-2 L/R; Du et al. 2020). Subsequently, several differences were reported when comparing sequences obtained from representatives of each clade using these primer sets. Differences in the genetic structure of the partial *MAT1-1-1* and *MAT1-2-1* sequences obtained using these primers were observed between the black and yellow clades. The length of the genes differed between both clades: *MAT1-1-1* was 729–736 bp in length in black morels and 708 bp in yellow morels, while *MAT1-2-1* was 398–408 bp in black morels and 869–880 bp in yellow morels (Du et al. 2017, 2020). In addition, there are notable differences in the genetic architecture of the mating-type regions of black and yellow morels, as *MAT1-1-10* was observed in Mes-20 (Esculenta clade) (Chai et al. 2019) but both *MAT1-1-10* and *MAT1-1-11* were present within the mating region in *M. importuna* (Elata clade) (Chai et al. 2019).

Until now, these previously published primers were mainly used to investigate mating types in morel strains from an Asian origin (Du et al. 2017, 2020). However, due to the diverging evolutionary histories of morel species from different geographical regions (O’Donnell et al. 2011), their performance in populations of other origins might vary and thus needs to be assessed. In thus study, we aimed at evaluating the performance of primers designed to amplify partial regions of *MAT1-1-1* and﻿ *MAT1-2-1* genes specifically in the Elata (Du et al. 2017) and Esculenta (Du et al. 2020) clades using in-silico methods. For this, custom hidden Markov model (HMM) profiles were designed to identify putative *MAT1-1-1* and *MAT1-2-1* genes in *Morchella* genomes. Additionally, our investigations considered genomic context when identifying putative mating-type regions, specifically the location of potential *MAT* genes relative to the *APN2* and *SLA2* genes, which often flank the mating-type region (Robinson et al. 2019). The NCBI Primer-BLAST tool and other alignment-based methods were used to estimate the efficiency of the previously published primer sets in amplifying the intended mating-type genes from *Morchella* genomes. These investigations provided further support that the Elata-specific *MAT1-2-1* primer pair would correctly amplify the proper *MAT* gene in the Elata clade and in the Esculenta clade. On the other hand, all other examined primers have the potential to amplify incorrect off-targets in diverse *Morchella* genomes and do not function as intended. This was further confirmed with PCR experiments utilizing the published primer sequences. This work highlights important considerations when designing and evaluating primers for the characterization of diverse fungi and provides insights as to how mating-type specific primers can be properly developed using available genomic resources.

## Main text

### In silico analysis of mating-type gene primers for *Morchella*

Two sets of Hidden Markov model (HMM) profiles were created for both *MAT1-1-1* and *MAT1-2-1*. One set was created using protein sequences from several *Tuber* and *Morchella* species, and the other set was created using non-Pezizomycetes sequences from diverse ascomycetes. Details of these HMM profiles and their summary statistics can be found in Additional file 1. Amino-acid alignments for the creation of the HMM profiles were generated using Clustal Omega (Sievers et al. 2011). The non-Pezizomycetes HMM profile included sequences from highly studied taxa that helped establish canonical models for mating-type genes, such as *Neurospora* and *Magnaporthe*, making them generally more trustworthy than largely unverified sequences from *Tuber* or *Morchella*. Additionally, published sequences for *Morchella* and *Tuber* were quite limited in number. These HMM profiles were used to search (hmmsearch) predicted protein sequences from *M. importuna* strain SCYDJ1-A1 (Morimp1) and *M. importuna* strain CCBAS932 (Morco1) (Additional file 2). The genome assembly and annotated protein sequences for these *M. importuna* isolates were obtained from the Joint Genome Institute (JGI) MycoCosm portal (https://mycocosm.jgi.doe.gov/mycocosm/home). Putative mating-type genes identified by these HMM profiles were further analyzed by comparing their position relative to the *APN2* and *SLA2* genes within the genome for each isolate examined. The *APN2* and *SLA2* genes were identified in the *M. importuna* genomes through homology-based searches using genes from *Neurospora* as a reference (NCBI accessions: XP_964240.1 and ESA43843.1). Putative mating-type genes identified in the HMM searches that were also located between *APN2* and *SLA2* loci were considered to have the strongest support and selected for all downstream analyses. Nucleotide and amino acid sequences for both the putative *M. importuna* mating-type genes and the region containing the mating-type locus and flanking genes can be found in Additional file 3 along with their coordinates in each genome assembly and other relevant identifiers. The nucleotide sequences for both the putative *MAT1-1-1* and *MAT1-2-1* genes from *M. importuna* were aligned to the genome assembly of *M. crassipes* strain M10 (NCBI accession: GCA_009192285.1) and *M. eximia* strain MG90 (GCA_003314645.1) using blastn. Nucleotide sequences of *APN2* and *SLA2* obtained from *M. importuna* were also used to identify homologs in these assemblies using blastn. The nucleotide sequence for the putative *MAT1-1-1* gene identified in the *M. crassipes* strain M10 assembly and the putative *MAT1-2-1* gene identified in the *M. eximia* strain MG90 can be found along with their coordinates in Additional file 3.

The NCBI Primer-BLAST tool (https://www.ncbi.nlm.nih.gov/tools/primer-blast/index.cgi) was used to screen the genome assemblies of *M. eximia* strain MG90 (GCA_003314645.1) and *M. crassipes* strain M10 (GCA_009192285.1). All default parameters were used in screens performed with Primer-BLAST and the primer pairs used in each screen were entered as the forward primer (L) and reverse primer (R). Previously published primers for mating-type genes were also aligned to the *Morchella* genome assemblies investigated in this work using blastn (-task blastn-short). All alignments involving nucleotide sequences of amplicons, putative mating-type genes, and/or *Morchella* genome assemblies were performed using blastn.

### Evaluating the design of previously published mating-type primers for Elata clade

In silico analysis of the published PCR primers designed to amplify *MAT1-1-1* and *MAT1-2-1* genes in *Morchella* were performed. First, custom designed HMM profiles developed for this work were used to screen annotated genome assemblies of two *M. importuna* isolates (Elata clade) obtained from the JGI MycoCosm portal. These screens identified a putative *MAT1-1-1* gene in one of the isolates (CCBAS932/Morco1) and a putative *MAT1-2-1* gene in the other isolate (SCYDJ1-A1/Morimp1). The putative mating-type regions in these *M. importuna* genomes are highly conserved other than for the loci encoding the mating-type genes (Additional file 4). These putative mating-type gene sequences were then used to genotype the genome of *M. eximia* strain MG90 (GCA_003314645.1), which was the assembly used to generate the Elata-specific primers (Du et al. 2017). A high coverage (98%) and identity (94.77%) alignment was found between the putative *MAT1-2-1* gene sequence and the *M. eximia* genome assembly, while the alignment with the putative *MAT1-1-1* gene sequence had lower coverage (2%) and identity (81.40%). These results appear to indicate that the *M. eximia* reference genome used to design both the *MAT1-1-1* and *MAT1-2-1* primers is heterothallic and harbors only the MAT1-2 idiomorph. Next, we aligned the primers against this *M. eximia* genome using the NCBI Primer-BLAST tool. Three amplicon products were predicted for the MAT1-1 primer set (MAT11L/R), none of which overlap with the mating-type region identified in the genomic screen. Two amplicon products which both overlap with the region identified in the genomic screen were predicted for the MAT1-2 primer set (MAT22L/R), but the product size was highly variable (Additional file 5) (502 and 2248 bp). Alignments between the MAT1-2 primer set and the *M. eximia* and *M. importuna* (Morimp1) genome assemblies indicated the top alignments overlapped with the putative mating-type regions identified in our genomic examinations. However, similar alignments between the MAT11 primer set and the *M. importuna* (Morco1) genome assembly yielded a relatively low identity score (65–70%) and only the MAT11L primer aligned to the putative mating type region identified (Additional file 6).

In addition to the in silico analysis, PCR experiments were performed with samples from a morel collection obtained in Switzerland. We compared amplicon sequences obtained from our *Morchella* isolates to those published by the authors of the primer sets (NCBI PopSet 1,213,383,383 and 1,213,383,466). The majority of our sequenced amplicon products obtained from members of the Elata clade using the MAT11 primer set were highly similar (> 98% coverage and identity) to those published by the authors. Alignments between our amplicon products and the putative MAT1-1-1 sequence obtained from *M. importuna* also showed high sequence similarity (90–97% identity). This was repeated for the MAT22 primer set with similar results from comparisons with the author published amplicon sequences (> 90% coverage and identity), but the comparisons with the putative MAT1-2-1 sequence from *M. importuna* revealed relatively lower sequence similarity (74–90% identity).

### Evaluating the design of previously published mating-type primers for Esculenta clade

In order to evaluate the primers for the Esculenta clade in a similar fashion, we first aligned the putative mating-type gene sequences from *M. importuna* to the genome of *M. crassipes* strain M10. An alignment (92% coverage and 73.25% identity) was found for the *MAT1-1-1* sequence, while no alignment was found for the *MAT1-2-1* sequence. This putative *MAT1-1-1* gene was located between the *APN2* and *SLA2* gene annotations. In a similar fashion to the investigation of the Elata clade primers, the EMAT primer sets were aligned to *M. crassipes* strain M10 genome assembly. A total of three amplicon products were predicted for the EMAT1-2 primer set (EMAT1-2 L/R) and no amplicon products were predicted for the EMAT1-1 primer set (EMAT1-1 L/R; Additional file 5). One of the three predicted amplicon products overlapped with the genome region that aligned with the putative *MAT1-1-1* sequence identified in the *M. importuna* alignment, while the other two were located on separate contigs (Additional file 5).

Alignments between the amplicon sequences we obtained and the published amplicon sequences from Du et al. (2020) (NCBI PopSet 1,809,496,744 and 1,809,496,908) generated using the same primers found no significant alignments for either the EMAT1-1 or EMAT1-2 primer set. Alignments between our amplicon products obtained using the EMAT1-1 primers and the NCBI nucleotide database yielded no significant similarity with any sequence, while the amplicon sequences we obtained from the EMAT1-2 primer sets had DNA-dependent RNA polymerase II (RPB2) sequences from *Morchella* as the top hits.

## Conclusion

The importance of sexual reproduction in the life-cycle of numerous species used for human consumption (e.g., *Morchella* spp.) (Pilz et al. 2007), medicine, or as insect pests biocontrol agents (e.g., *Cordyceps* sp.) (Zheng et al. 2013; Zou et al. 2019) makes a general understanding of fungal sexual reproduction relevant to many fields. This knowledge is particularly essential in conservation biology, where the potential for species invasiveness and genetic recombination between geographically isolated populations depends strongly on the possibility of genetic exchange and the formation of viable hybrids. In addition, the study of mating types also contributes to phylogenetic studies that aid in understanding the evolution of fungi (Du et al. 2017; Zou et al. 2019), as mating genes generally evolve more rapidly than non-mating ones, and can delimit species (Chai et al. 2019). In the specific case of morels, even though it has been possible to cultivate morels, the exact mechanisms underlying fruiting body formation are still not completely understood. It is generally assumed that two aspects are essential for the formation of fruiting-bodies: the intricate ecological requirements of the fungus, which include climatic, edaphic and biotic factors; as well as the possibility to achieve a sexual cycle through the appropriate encounter of compatible participants (Liu et al. 2018a). Morels were long considered as strictly heterothallic and thus, encounters of mycelia carrying the two opposite idiomorphs was necessary to produce fruiting bodies. However, recently, it has been shown that some species such as *M. importuna* can produce ascospores containing both mating types. These pseudohomothallic sexual spores give rise to fertile ascocarps and are easier to cultivate in comparison to heterothallic species or strains (Du et Yang 2021). Therefore, a reliable way to identify mating types in these fungi is necessary. For this purpose, primer pairs have been designed to rapidly and readily amplify by PCR mating-type genes *MAT1-1-1* and *MAT1-2-1* in black (Du et al. 2017) and yellow morels (Du et al. 2020). However, our analyses indicated that three out of four of those primer pairs are not reliable. The in silico analysis and the complementary in vitro confirmation presented here demonstrated that only the MAT22L/R was trustworthy as it did not lead to off-targets, which are problematic in the case of MAT11L/R. In contrast, the reported EMAT1-1 L/R and EMAT1-2 L/R primer sequences appear as inappropriate for assessing mating genotypes in *Morchella* since they target other genetic regions. For future studies, the in silico and experimental validation of other primer sets (Chai et al. 2017, 2019), or the design of new primer sets based on genomes from single-ascospore cultures genome is still needed to identify putative MAT idiomorphs and sexual reproduction strategies in Swiss morels.

## Supplementary Information

**Additional file 1**. Information in the sequences used for the generation of Hidden Markov model (HMM) profiles.

**Additional file 2**. Search of the Hidden Markov model (HMM) profiles in *M. importuna* strain SCYDJ1-A1 (Morimp1) and *M. importuna* strain CCBAS932 (Morco1).

**Additional file 3**. Nucleotide and amino acid sequences for the putative mating-type genes and the region containing the mating-type locus and flanking genes in the *Morchella* genomes investigated here.

**Additional file 4**. Comparisons of synteny in the putative mating-type regions between *M. importuna* strain SCYDJ1-A1 (Morimp1) and *M. importuna* strain CCBAS932 (Morco1) using the nucmer utility from the MUMmer package. The flanking regions are highly conserved, while the region containing the putative mating-type genes is dissimilar.

**Additional file 5**. Results of the primer search using NCBI Primer-BLAST tool.

**Additional file 6**. Results from blastn alignments between MAT primer sets and *Morchella* genomes. Assemblies’ alignments that overlap with putative mating type regions are highlighed in green. High identity alignments that do not overlap with putative mating type regions are highlighted in red.

## Data Availability

All data generated or analysed during this study are included in this published article and its supplementary information files S1 and S6.

## Abbreviations

HMM: Hidden Markov model
MAT: Mating type
RPB2: RNA polymerase II subunit
tntBLAST: Thermonucleotide BLAST

## References

[CR1] Arie T, Christiansen SK, Yoder OC, Turgeon BG (1997). Efficient cloning of ascomycete mating type genes by PCR amplification of the conserved MAT HMG box. Fungal Genet Biol.

[CR2] Casselton LA (2002). Mate recognition in fungi. Heredity.

[CR3] Chai H, Chen L, Chen W, Zhao Q, Zhang X, Su K, Zhao, (2017). Characterization of mating-type idiomorphs suggests that morchella importuna, Mel-20 and M Sextelata are heterothallic. Mycological Progress.

[CR4] Chai H, Chen W, Zhang X, Su K, Yongchang Zhao (2019). Structural variation and phylogenetic analysis of the mating-type locus in the genus morchella. Mycologia.

[CR5] Chai H, Liu P, Ma Y, Chen W, Tao N, Yongchang Zhao (2022). Organization and unconventional integration of the mating-type loci in Morchella species. J Fungi.

[CR6] Coppin E, Debuchy R, Arnaise S, Picard etM (1997). Mating types and sexual development in filamentous ascomycetes. Microbiol Mol Biol Rev.

[CR7] Du M, Schardl CL, Nuckles EM, Lisa J, Vaillancourt (2005). Using mating-type gene sequences for improved phylogenetic resolution of Collectotrichum species complexes. Mycologia.

[CR8] Du X-H, Zhao Q, Xia E-H, Gao L-Z (2017). et Franck Richard. Mixed-reproductive strategies, competitive mating-type distribution and life cycle of fourteen black morel species. Sci Rep.

[CR9] Du X-H, Wu D, Kang H, Wang H, Xu N, Li T, Keliang Chen (2020). Heterothallism and potential hybridization events inferred for twenty-two yellow morel species. IMA Fungus.

[CR10] Du X-H, Zhu L, Yang (2021). Mating systems in true morels (Morchella). Microbiol Mol Biol Rev.

[CR11] Liu Q, Ma H, Zhang Y, Caihong, Dong (2018). Artificial cultivation of true morels: current state, issues and perspectives. Crit Rev Biotechnol.

[CR12] Liu W, LianFu Chen, YingLi Cai, QianQian Zhang (2018). et YinBing Bian. Opposite polarity monospore genome De Novo sequencing and comparative analysis reveal the possible heterothallic life cycle of Morchella importuna. Int J Mol Sci.

[CR13] O’Donnell K, Rooney AP, Mills GL, Kuo M, Weber NS, Rehner SA (2011). Phylogeny and historical biogeography of true morels (Morchella) reveals an early cretaceous origin and high continental endemism and provincialism in the holarctic. Fungal Genet Biol.

[CR14] Pilz D, McLain R, Alexander S, Villarreal-Ruiz L, Berch S, Wurtz TL, Parks CG (2007). Ecology and management of morels harvested from the forests of Western North America. Pac Northwest Res Stn.

[CR15] Robinson AJ, Donald O, Natvig (2019). Diverse members of the Xylariales lack canonical mating-type regions. Fungal Genet Biol.

[CR16] Sievers F, Wilm A, Dineen D, Gibson TJ, Karplus K, Li W, Lopez R (2011). Fast, scalable generation of high-quality protein multiple sequence alignments using clustal omega. Mol Syst Biol.

[CR17] Wilken P, Markus ET, Steenkamp MJ, Wingfield Z, Wilhelm de Beer D, Wingfield (2017). Which MAT gene? Pezizomycotina (Ascomycota) mating-type gene nomenclature reconsidered. Fungal Biol Rev.

[CR18] Wilson AM, Markus Wilken P, Magriet A, van der Nest ET, Steenkamp MJ, Wingfield D, Wingfield (2015). Homothallism: an umbrella term for describing diverse sexual behaviours. IMA Fungus.

[CR19] Zheng P, Xia Y, Zhang S, Chengshu, Wang (2013). Genetics of cordyceps and related fungi. Appl Microbiol Biotechnol.

[CR20] Zou J, Zeng T-T, He Z-M, Zhang P, Zuo-Hong C (2019). Cloning and analysis of ophiocordyceps Xuefengensis mating type (MAT) loci. FEMS Microbiol Lett.

