# Supplementary material for: Importance of appropriate genome information for the design of mating type primers in black and yellow morel populations
Source: IMA Fungus. 2022 Aug 22;13:14. doi: 10.1186/s43008-022-00101-6 (PMC9394083; doi:10.1186/s43008-022-00101-6)
Supplement: Supplementary file 1 — Additional file 1. Information in the sequences used for the generation of Hidden Markov model (HMM) profiles. [file 43008_2022_101_MOESM1_ESM.docx]

**Importance of appropriate genome information for the design of mating type primers in black and yellow morel populations**

**Supplementary File S1.** Information in the sequences used for the generation of Hidden Markov model (HMM) profiles

*Tuber*/*Morchella* MAT1-1-1 sequences:

- PUU82705.1 mating type protein MAT1-1-1 [Tuber borchii]
- ADU56595.1 mating type protein MAT1-1-1 [Tuber melanosporum]
- AHE80940.1 MAT1-1-1 [Tuber indicum]
- AVI61100.1 mating type protein MAT1-1-1, partial [Morchella purpurascens]
- AVI61099.1 mating type protein MAT1-1-1 [Morchella septimelata]
- AVI61084.1 mating type protein MAT1-1-1 [Morchella sp. Mes-20]

*Tuber*/*Morchella* MAT1-2-1 sequences:

- AVI60844.1 mating type protein MAT1-2-1 [Morchella crassipes]
- AVI61124.1 mating type protein MAT1-2-1 [Morchella galilaea]
- AIU38078.1 Mat1-2-1 [Tuber borchii]

Non-Pezizomycetes MAT1-1-1 sequences:

- AAC37478.1 mating type protein [Neurospora crassa] A-1
- BAC65091.1 MAT1-1-1 [Magnaporthe grisea]
- AMP43945.1 MAT 1-1-1 [Fusarium graminearum]
- KAA8630680.1 MAT1-1-1 [Sordaria macrospora]
- BAC65083.1 MAT1-1-1 [Pyricularia grisea]
- RKF71893.1 Mating type protein A-1 [Golovinomyces cichoracearum]
- AGW27562.1 MAT1-1-1 [Tolypocladium inflatum]
- ACR78244.1 MAT1-1-1 [Trichoderma reesei]
- OAA38729.1 mating-type protein MAT 1-1-1 [Beauveria brongniartii RCEF 3172]

Non-Pezizomycetes MAT1-2-1 sequences:

- AAA33598.2 mating type a-1 protein [Neurospora crassa]
- BAC65090.1 MAT1-2-1 [Magnaporthe grisea]
- ALD16247.1 mat a-1 [Thermothelomyces hinnuleus]
- AIG95712.1 MAT1-2-1 [Pseudogymnoascus destructans]
- AGS32062.1 MAT1-2-1 [Leptographium profanum]
- ADB96183.1 MAT-1-2 [Ophiostoma himal-ulmi]
- AGS32056.1 MAT1-2-1 [Leptographium procerum]
- OAA63301.1 mating type protein 1-2-1 [Sporothrix insectorum RCEF 264]
- AST15023.1 mat1-2-1 [Trichoderma spinulosum]
- XP_014174596.1 mating type protein 1-2-1 [Grosmannia clavigera kw1407]
- AFQ62783.1 MAT1-2-1 [Hymenoscyphus albidus]

HMM Summary Statistics

| HMM profile | nseq | eff_nseq | M | relent | info | p relE | compKL |
| --- | --- | --- | --- | --- | --- | --- | --- |
| MAT1-1-1 *Tuber*/*Morchella* | 6 | 0.74 | 456 | 0.59 | 0.57 | 0.51 | 0.01 |
| MAT1-2-1 *Tuber*/*Morchella* | 3 | 0.65 | 376 | 0.59 | 0.58 | 0.51 | 0.01 |
| MAT1-1-1 Non-Pezizomycetes | 9 | 1.61 | 366 | 0.59 | 0.56 | 0.50 | 0.02 |
| MAT1-2-1 Non-Pezizomycetes | 11 | 1.69 | 347 | 0.59 | 0.56 | 0.45 | 0.03 |
|  |  |  |  |  |  |  |  |

Descriptions for hmmstat output fields can be found at: <http://eddylab.org/software/hmmer/Userguide.pdf>
