# Supplementary material for: Importance of appropriate genome information for the design of mating type primers in black and yellow morel populations
Source: IMA Fungus. 2022 Aug 22;13:14. doi: 10.1186/s43008-022-00101-6 (PMC9394083; doi:10.1186/s43008-022-00101-6)

**Importance of appropriate genome information for the design of mating type primers in black and yellow morel populations**

**Supplementary File S2.** Search of the Hidden Markov model (HMM) profiles in *M. importuna* strain SCYDJ1-A1 (Morimp1) and *M. importuna* strain CCBAS932 (Morco1)

Hmmsearch of *M. importuna* strain CCBAS932


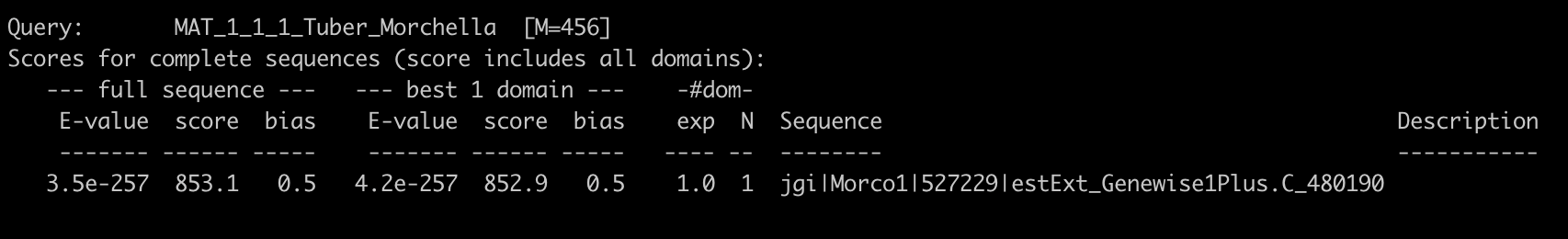


Hmmsearch of *M. importuna* strain SCYDJ1-A1


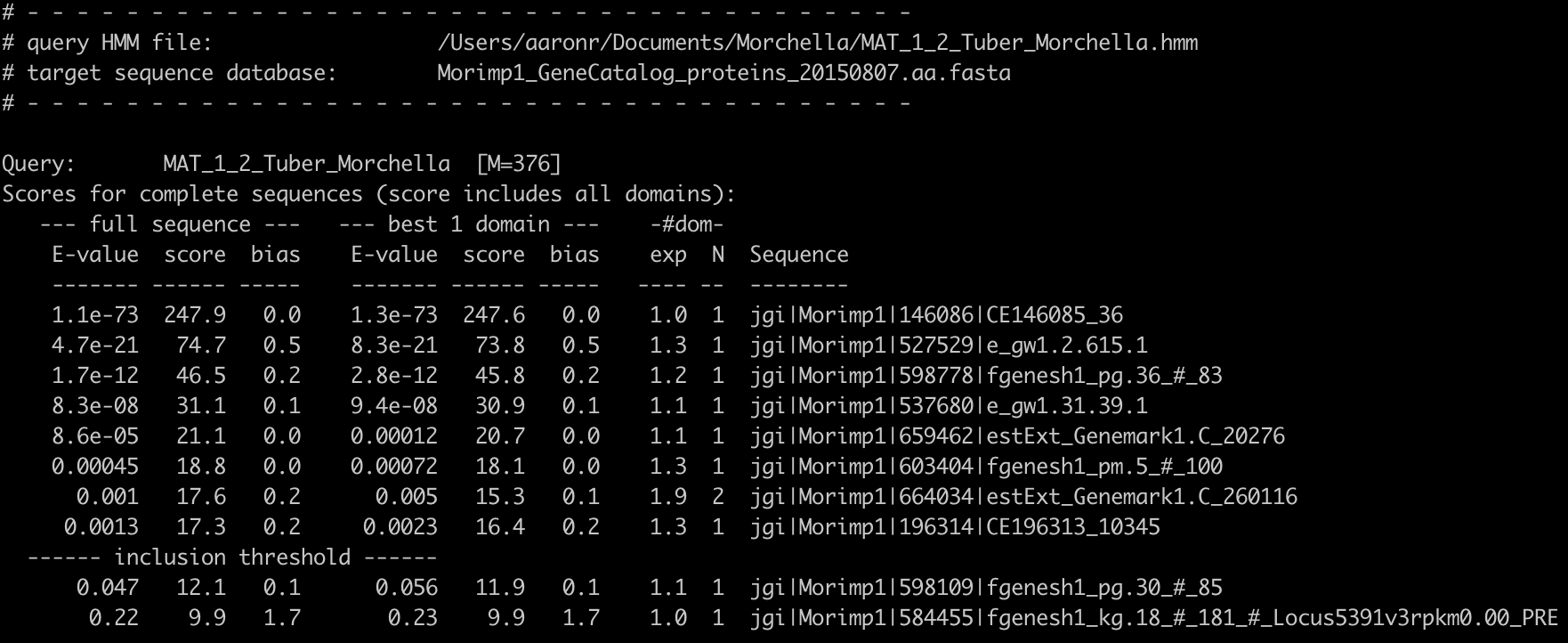

Supplement: Supplementary file 2 — Additional file 2. Search of the Hidden Markov model (HMM) profiles in M. importuna strain SCYDJ1-A1 (Morimp1) and M. importuna strain CCBAS932 (Morco1). [file 43008_2022_101_MOESM2_ESM.docx]
