# Supplementary material for: Importance of appropriate genome information for the design of mating type primers in black and yellow morel populations
Source: IMA Fungus. 2022 Aug 22;13:14. doi: 10.1186/s43008-022-00101-6 (PMC9394083; doi:10.1186/s43008-022-00101-6)

**Importance of appropriate genome information for the design of mating type primers in black and yellow morel populations**

**Supplementary File S4.** Comparisons of synteny in the putative mating-type regions between M. importuna strain SCYDJ1-A1 (Morimp1) and M. importuna strain CCBAS932 (Morco1) using the nucmer utility from the MUMmer package. The flanking regions are highly conserved, while the region containing the putative mating-type genes is dissimilar.


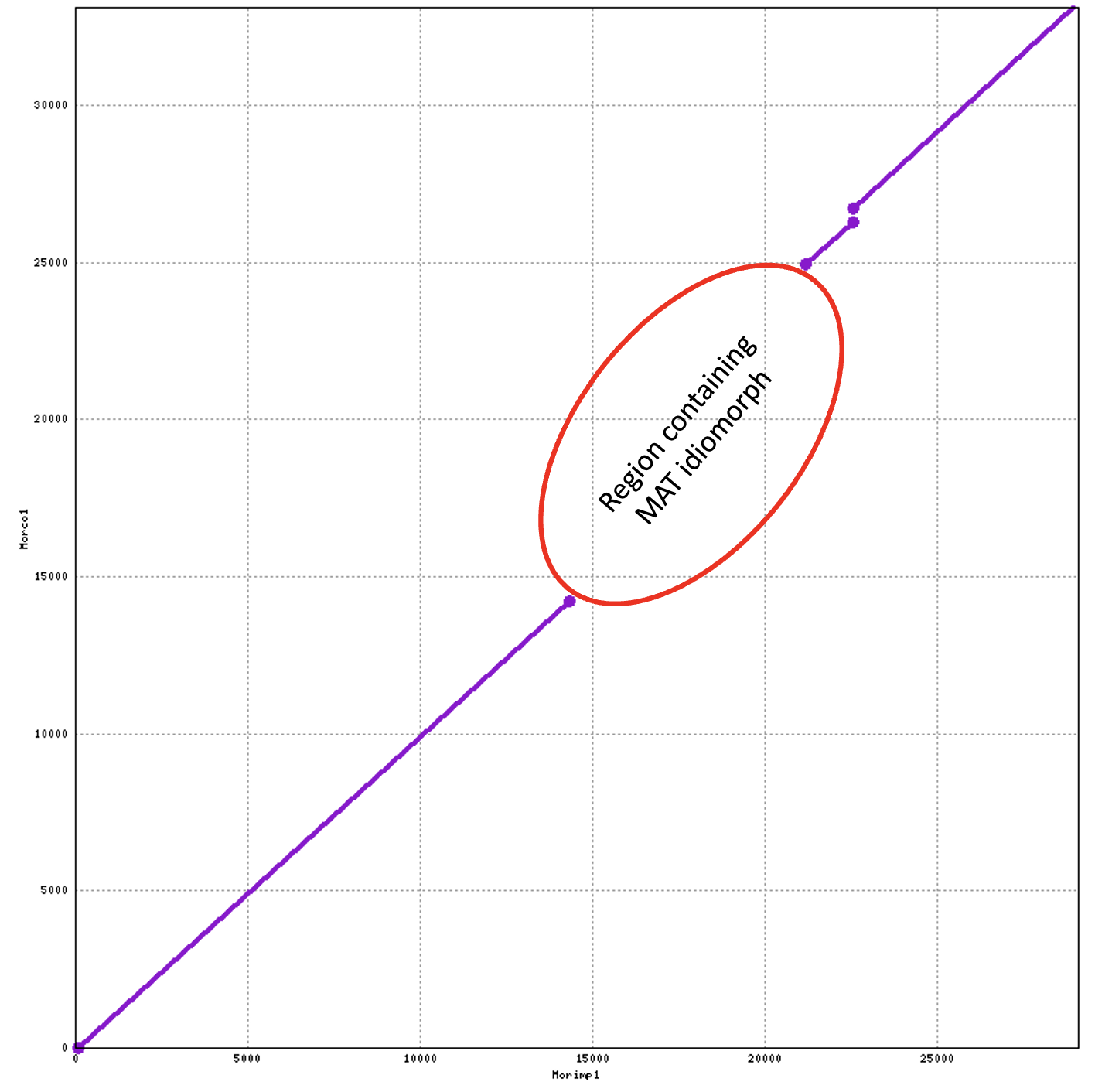

Supplement: Supplementary file 4 — Additional file 4. Comparisons of synteny in the putative mating-type regions between M. importuna strain SCYDJ1-A1 (Morimp1) and M. importuna strain CCBAS932 (Morco1) using the nucmer utility from the MUMmer package. The flanking regions are highly conserved, while the region containing the putative mating-type genes is dissimilar. [file 43008_2022_101_MOESM4_ESM.docx]
