# Supplementary material for: Importance of appropriate genome information for the design of mating type primers in black and yellow morel populations
Source: IMA Fungus. 2022 Aug 22;13:14. doi: 10.1186/s43008-022-00101-6 (PMC9394083; doi:10.1186/s43008-022-00101-6)

**Importance of appropriate genome information for the design of mating type primers in black and yellow morel populations**

**Supplementary File S5.** Results of the primer search using NCBI Primer-BLAST tool

MAT11 primer set from Du et al. 2017 Primer-BLAST results for *M. eximia* strain MG90


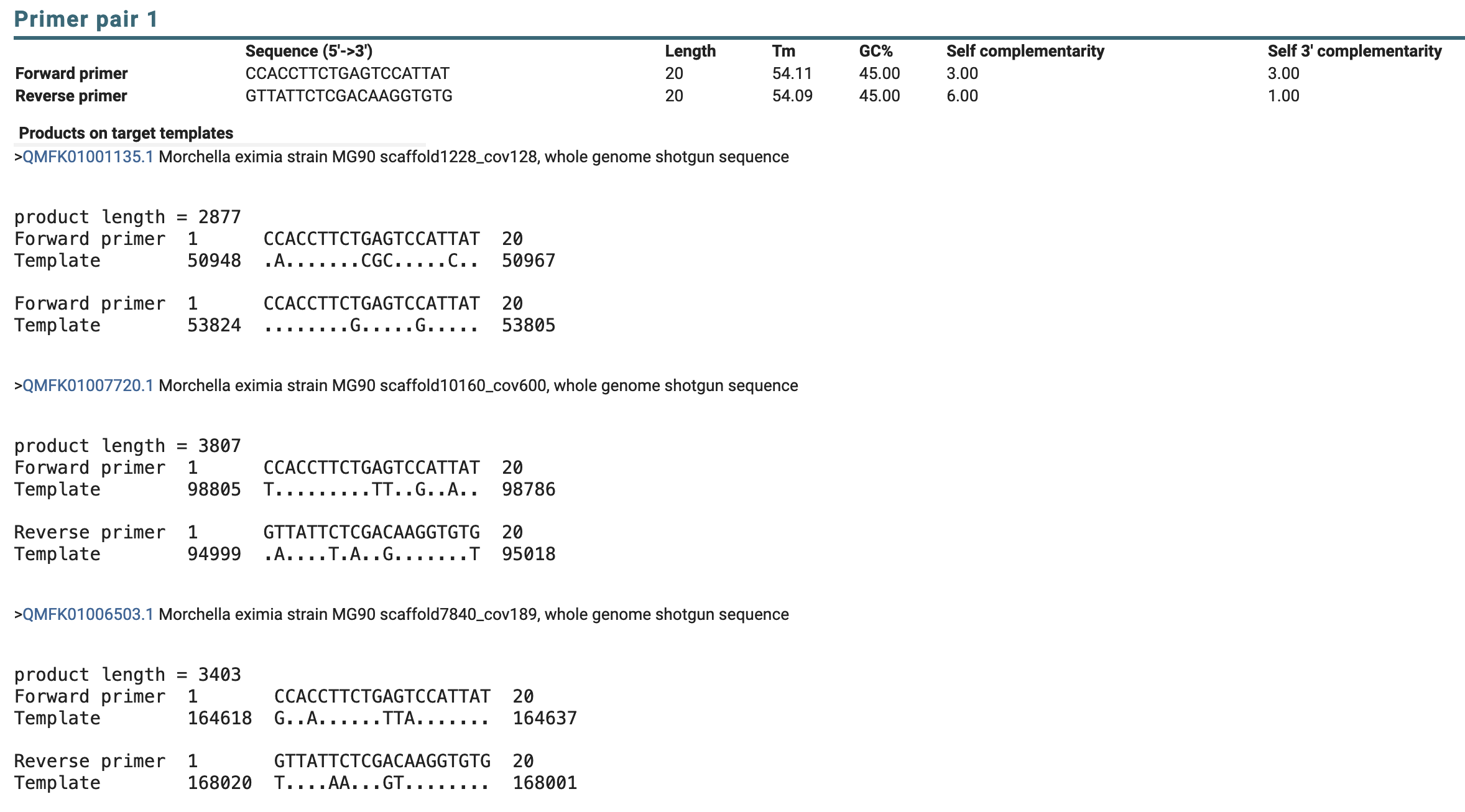


MAT22 primer set from Du et al. 2017 Primer-BLAST results for *M. eximia* strain MG90


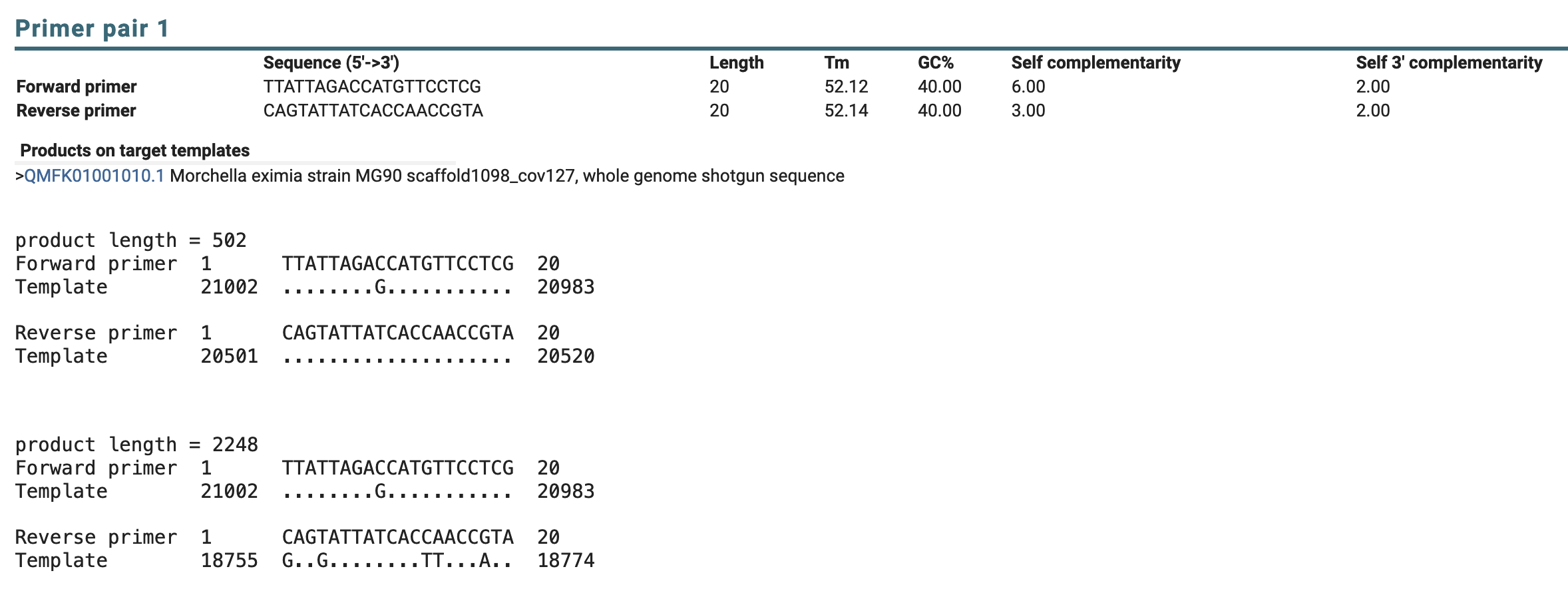


EMAT1-1 primer set from Du et al. 2020 Primer-BLAST results from *M. crassipes* strain M10


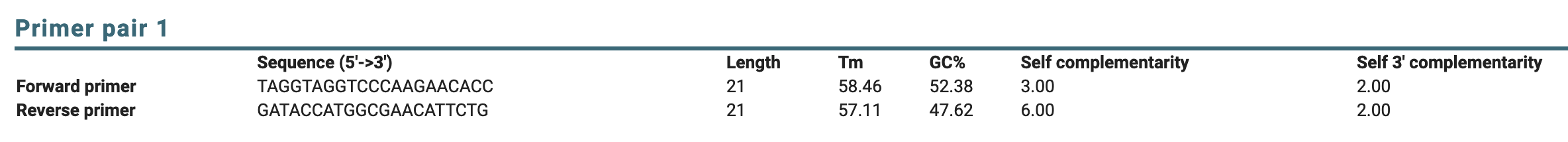


EMAT1-2 primer set from Du et al. 2020 Primer-BLAST results from *M. crassipes* strain M10


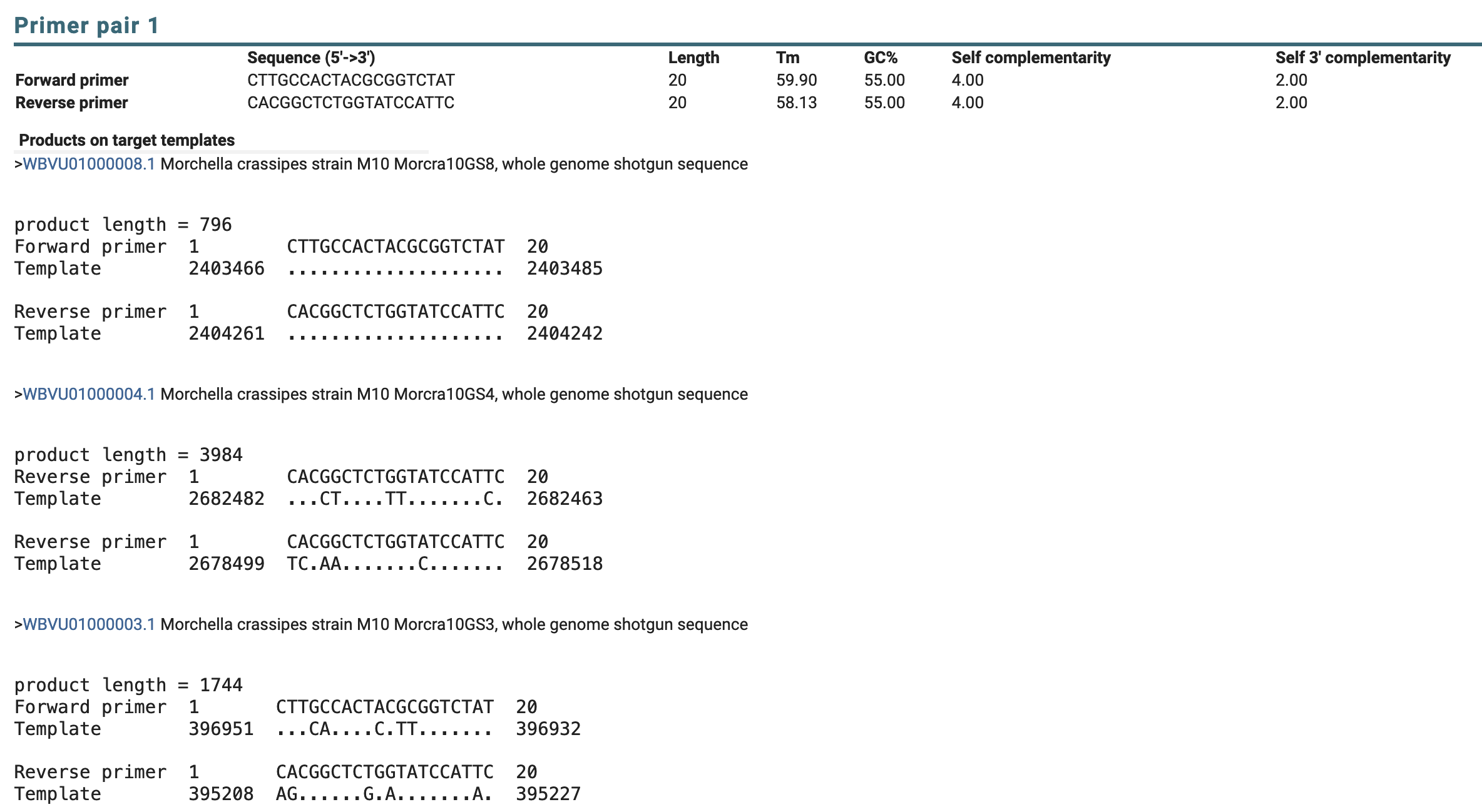

Supplement: Supplementary file 5 — Additional file 5. Results of the primer search using NCBI Primer-BLAST tool. [file 43008_2022_101_MOESM5_ESM.docx]
